# Cardiac Overexpression of Constitutively Active Galpha q Causes Angiotensin II Type1 Receptor Activation, Leading to Progressive Heart Failure and Ventricular Arrhythmias in Transgenic Mice

**DOI:** 10.1371/journal.pone.0106354

**Published:** 2014-08-29

**Authors:** Naoko Matsushita, Toshihide Kashihara, Hisashi Shimojo, Satoshi Suzuki, Tsutomu Nakada, Yasuchika Takeishi, Ulrike Mende, Eiichi Taira, Mitsuhiko Yamada, Atsushi Sanbe, Masamichi Hirose

**Affiliations:** 1 Department of Molecular and Cellular Pharmacology, Iwate Medical University School of Pharmaceutical Sciences, Shiwa, Iwate, Japan; 2 Department of Pharmacology, Iwate Medical University School of Medicine, Shiwa, Iwate, Japan; 3 Department of Molecular Pharmacology, Shinshu University School of Medicine, Matsumoto, Nagano, Japan; 4 Department of Pathology, Shinshu University School of Medicine, Matsumoto, Nagano, Japan; 5 Department of Cardiology and Hematology, Fukushima Medical University, Fukushima, Fukushima, Japan; 6 Cardiovascular Research Center, Division of Cardiology, Rhode Island Hospital & The Alpert Medical School of Brown University, Providence, Rhode Island, United States of America; 7 Department of Pharmacotherapeutics, Iwate Medical University School of Pharmaceutical Sciences, Shiwa, Iwate, Japan; Rutgers New Jersey Medical School, United States of America

## Abstract

**Background:**

Transgenic mice with transient cardiac expression of constitutively active Galpha q (Gα_q_-TG) exhibt progressive heart failure and ventricular arrhythmias after the initiating stimulus of transfected constitutively active Gα_q_ becomes undetectable. However, the mechanisms are still unknown. We examined the effects of chronic administration of olmesartan on heart failure and ventricular arrhythmia in Gα_q_-TG mice.

**Methodology/Principal Findings:**

Olmesartan (1 mg/kg/day) or vehicle was chronically administered to Gα_q_-TG from 6 to 32 weeks of age, and all experiments were performed in mice at the age of 32 weeks. Chronic olmesartan administration prevented the severe reduction of left ventricular fractional shortening, and inhibited ventricular interstitial fibrosis and ventricular myocyte hypertrophy in Gα_q_-TG. Electrocardiogram demonstrated that premature ventricular contraction (PVC) was frequently (more than 20 beats/min) observed in 9 of 10 vehicle-treated Gα_q_-TG but in none of 10 olmesartan-treated Gα_q_-TG. The collected QT interval and monophasic action potential duration in the left ventricle were significantly shorter in olmesartan-treated Gα_q_-TG than in vehicle-treated Gα_q_-TG. CTGF, collagen type 1, ANP, BNP, and β-MHC gene expression was increased and olmesartan significantly decreased the expression of these genes in Gα_q_-TG mouse ventricles. The expression of canonical transient receptor potential (TRPC) 3 and 6 channel and angiotensin converting enzyme (ACE) proteins but not angiotensin II type 1 (AT_1_) receptor was increased in Gα_q_-TG ventricles compared with NTG mouse ventricles. Olmesartan significantly decreased TRPC6 and tended to decrease ACE expressions in Gα_q_-TG. Moreover, it increased AT_1_ receptor in Gα_q_-TG.

**Conclusions/Significance:**

These findings suggest that angiotensin II type 1 receptor activation plays an important role in the development of heart failure and ventricular arrhythmia in Gα_q_-TG mouse model of heart failure.

## Introduction

Our previous study showed that transient expression of a constitutively active the GTP-binding protein α_q_ subunit in hearts of transgenic mice (Gα_q_-TG mice) is sufficient to induce cardiac hypertrophy and heart failure (HF) [1]. In fact, although the Gα_q_ protein decreases at 4 weeks and is undetectable until 10 weeks, the mice develop cardiac hypertrophy and dilatation, leading to HF until 16 to 32 weeks of age [1]–[4]. When the cardiac hypertrophy and dilatation develop, endogenous but not transfected Gα_q_ rises in the heart. Basal and G_q_-coupled receptor agonist stimulated activity of phospholipase Cß (PLCß), leading to generatiton of inositol trisphosphate (IP_3_) and diacylglycerol (DAG), which is elevated in ventricles at 10 week age in Gα_q_-TG mice, presumably at least in part because of the rise in endogenous Gα_q_ [1], [5]. Therefore, the pathological changes initiated by early transient constitutively active Gα_q_ expression may be maintained by multiple and persistent changes in signal transduction pathways [1], [5]. Our more recent studies demonstrated that diacylglycerol kinase zeta, which catalyzes DAG, rescues HF [2] and inhibited atrial [3] and ventricular [4] arrhythmias in Gα_q_-TG mice, suggesting that DAG plays a critical role in the development of cardiac hypertrophy and HF in this mouse model. However, it is still unknown what factors act upstream of DAG. It is well known that the renin-angiotensin system, which increases the level of DAG, plays a critical role in the development of cardiac hypertrophy and HF [6]–[8]. We hypothesized that the renin-angiotensin system plays an important role in the development of cardiac hypertrophy and HF in this transgenic mouse model after the initiating stimulus of transfected constitutively active Gα_q_ becomes undetectable. Olmesartan is an angiotensin II type 1 receptor antagonist, which can inhibit angiotensin II-induced cardiac remodeling and HF [9], [10]. In the present study, therefore, we investigated the inhibitory effects of olmesartan on ventricular remodeling, leading to HF and ventricular arrhythmias in Gα_q_-TG mice.

## Materials and Methods

### Ethics

This study was carried out in strict accordance with the recommendations in the Guide for the Care and Use of Laboratory Animals of the National Institutes of Health. This study was approved by the Animal Care Committee of the Iwate Medical University and Shinshu University. The protocol was approved by the Committee on the Ethics of Animal Experiments of the Iwate Medical University (Permit Number: 22–39) and the Shinshu University (Permit Number: 200044). All surgery was performed under sodium pentobarbital anesthesia, and all efforts were made to minimize suffering.

### Experimental Animals

A transgenic mouse (Gα_q_-TG mouse) with transient, modest expression of HAα*_q_ was used [1]. The genotypes of the non-transgenic (NTG) and Gα_q_-TG mice were identified by polymerase chain reaction (PCR) with the use of tail genomic DNA as a template, as previously reported [1]. Our previous studies demonstrated that Gα_q_-TG mice developed HF but not ventricular arrhythmias at the age of 16 weeks, whereas they developed ventricular arrhythmias by 32 weeks [4]. We measured the systemic blood pressure and heart rate using the tail-cuff method. (BP-98A Softron, Tokyo, Japan) and demonstrated that olmesartan at a dose of 1 mg/kg/day did not decrease the systemic blood pressure (Table 1). Therefore, to examine the effects of chronic olmesartan administration on HF and ventricular arrhythmias, olmesartan (1 mg/kg/day) was orally administered to Gα_q_-TG mice from 6 to 32 weeks of age. All experiments were performed in 32-week-old mice. As described in detail previously [11], all mice were anesthetized with sodium pentobarbital (30 mg/kg) applied intraperitoneally, and the adequacy of anesthesia was monitored by observing heart rate and the frequency and the degree of motion of the sternum as well as movement of the extremities.

**Table 1:** Systemic blood pressure (BP) and heart rates in NTG, Gα_q_-TG, and Gα_q_-TG+olmesartan mice.

Parameters	NTG	Gα_q_-TG	Gα_q_-TG+olmesartan
HR (beats/min)	613±23	566±45	651±20
SBP (mmHg)	102±6	94±3	103±3
DBP (mmHg)	62±3	60±4	64±2
MBP(mmHg)	75±4	72±5	77±1

Data are the mean ± SE obtained from 7 mice for each group. SBP, systolic BP; DBP, diastric BP; MBP, mean BP.

### Echocardiography

Vehicle-treated NTG, vehicle-treated Gα_q_-TG, and olmesartan-treated Gα_q_-TG mice (n  = 7 each) were anesthetized, and cardiac function was assessed by echocardiography (GE Yokogawa Medical System, Tokyo, Japan). As described in detail previously [12], the level of the papillary muscles along the short axis was used to view heart. The average of three consecutive beats in M-mode tracings was used to measure the following parameters: interventricular septum thickness, left ventricular end-diastolic dimension (LVEDd), end-systolic dimension (LVESd), and fractional shortening (LVFS), which was calculated as follows: (LVEDd - LVESd)/LVEDd×100%.

### Electrocardiography (ECG) and Electrophysiological Measurement

Vehicle-treated NTG, vehicle-treated Gα_q_-TG, and olmesartan-treated Gα_q_-TG mice (n = 7 each) were anesthetized with sodium pentobarbital (30 mg/kg) applied intraperitoneally. Electrocardiography (ECG) lead II was recorded for 10 min in all mice. As described in detail previously [11], surface ECG was recorded and filtered (0.1 to 300 Hz), digitized with 12-bit precision at a sampling rate of 1000 Hz per channel (Microstar Laboratories Inc., Bellevue, WA, USA), transmitted into a microcomputer and saved on a CD-ROM.

In all mice examined, P, PR, QRS complex, QT, and RR intervals were measured from ECG lead II. The number of premature ventricular contractions (PVCs) per minute was calculated from ECG lead II. A high incidence of PVCs (High PVC) was defined as more than 20 beats/min of PVC.

### Gross Anatomy and Histology

After vehicle-treated NTG, vehicle-treated Gα_q_-TG, and olmesartan-treated Gα_q_-TG mice (n = 10 each) were anesthetized, hearts were quickly excised. To examine gross anatomy and histology, the heart preparation was prepared. As described in detail previously [11], the hearts were fixed with a 30% solution of formalin in phosphate-buffered saline at room temperature for more than 24 hours, embedded in paraffin, and then cut serially from the apex to the base. Six sections were stained with hematoxylin/eosin or Masson’s trichrome for histopathological analysis. To measure the cross-sectional diameter of cardiomyocytes, the diameter of at least 20 cardiomyocytes in each section was measured using the image analyzing software MacSCOPE (MITANI Corporation, Tokyo) on a Macintosh computer. The measurements were performed on 3 sections in each preparation and averaged. The degree of fibrosis was assessed by digital microscopic images taken from the sections stained with Masson’s trichrome stain using light microscopy with a digital camera system. As described in detail previously [3], the measurements were performed on 3 images from different parts of the left ventricle in each preparation. The fibrosis fraction was obtained by calculating the ratio of total connective area to total myocardial area from 3 images in each preparation.

### Western Blot Analysis

The ventricular myocardium of anesthetized NTG, vehicle-treated Gα_q_-TG, and olmesartan-treated Gα_q_-TG mice (n = 6 each) was prepared to extract the total protein using a lysis buffer (Cell Signaling Technology, Inc., Danvers, MA). The protein expression of canonical transient receptor potential (TRPC) and angiotensin-converting enzyme (ACE) isoforms was examined. As described in detail previously [11], protein concentrations were assayed, and equal amounts of the proteins were subjected to 10% SDS-PAGE and transferred to PVDF membranes. To ensure equivalent protein loading and to verify efficient protein transfer, membranes were stained with Ponceau S before incubating with primary isoform-specific antibodies against TRPC isoforms (TRPC 3 and 6; SIGMA, St. Louis, MO), ACE isoforms (ACE and ACE2; SIGMA, St. Louis, MO) and actin. [13] Immunoreactive bands were detected with an ECL kit (Amersham Biosciences Corp., Piscataway, NJ). The densitometric intensity of bands representing TRPC and ACE isoforms was normalized to that of actin. The protein expression levels of angiotensin II type 1 (AT_1_) receptor in the ventricular myocardium of anesthetized NTG, vehicle-treated Gα_q_-TG, and olmesartan-treated Gα_q_-TG mice (n = 6 each) were also examined, as described in detail previously [14]–[15]. Western blot analyses were performed using anti-GAPDH antibody (Chemicon International, Temecula, CA, USA) and anti-AT_1_ receptor antibody (Santa Cruz Biotechnology, Dallas, TX, USA).

### Quantification of mRNA by Real-Time PCR

Total RNA was prepared from the ventricular myocardium of anesthetized NTG, vehicle-treated Gα_q_-TG and olmesartan-treated Gα_q_-TG mice (n = 7 each) with NueleoSpin RNA II (TAKARA Co. Ltd., Tokyo, Japan) according to the manufacturer’s instructions. The mRNA levels of atrial natriuretic factor (ANF), B-type natriuretic peptide (BNP), β-myosin heavy chain (β-MHC), connective tissue growth factor (CTGF), collagen type 1, and acidic ribosomal protein P0 (ARPP0) were examined. As described in detail previously [11], one microgram of total RNA was used as a template for reverse transcription with the SuperScript III First-Strand synthesis system for qRT-PCR (Invitrogen, Carlsbad, CA). Partial cDNA fragments of ANF, BNP, β-MHC, CTGF, collagen type 1, and ARPP0 were amplified from the heart cDNA by PCR with DNA polymerase Fast SYBR Green Master Mix (Takara Bio, Shiga, Japan) to generate a standard curve for mRNA quantification. Real-time PCR was performed with an ABI Step One Real-Time PCR System (Applied Biosystems, Foster City, CA). The PCR mixture (10 µl) contained Fast SYBR Green Master (Mix) (Roche Diagnostics), standard cDNA (5×10^2^ ng per reaction), and 200 nM forward and reverse primers. All primers used are listed in Table S1. The expression of each gene was normalized to that of ARPP0 mRNA because the expression of ARPP0 mRNA was most consistent among the groups. The specificity of the method was confirmed by dissociation analysis according to the instructions supplied by Applied Biosystems.

### Monophasic action potential (MAP) measurement

Vehicle-treated NTG, vehicle-treated Gα_q_-TG, and olmesartan-treated Gα_q_-TG mice (n = 6 each) were anesthetized with sodium pentobarbital (30 mg/kg) applied intraperitoneally, and then treated with sodium heparin (500 USP units/kg i.v.). After the hearts were quickly excised, we connected them to a Langendorff apparatus. We used a polyterafluoroethylene-coated silver bipolar electrode to stimulate the epicardial surface of the left ventricle at the twice diastolic threshold current with a duration of 1 ms. As described in detail previously [11], to measure the monophasic action potential (MAP) duration, we put on MAP electrode on the epicardial surface of the posterior left ventricle. MAPs were recorded for 5 sec at a basic cycle length of 200 ms. Each heart preparation was perfused under constant pressure conditions (65 mmHg) with oxygenated (95% oxygen, 5% CO_2_) Tyrode’s solution containing, in mM: NaCl, 141.0; KCl, 5.0; CaCl_2_, 1.8; NaHCO_3_, 25.0; MgSO_4_, 1.0; NaH_2_PO_4_, 1.2; HEPES, 5; and dextrose, 5.0 (pH of 7.4 at 36±1°C). The MAP signals were filtered (0.3 to 300 Hz), amplified (1,000×), and recorded. As described in detail previously [11], the MAP duration was calculated from MAP signals of all Langendorff hearts.

### Data Analysis

All data are shown as the mean ± SE. The statistical analysis of multiple comparisons of data was calculated using An analysis of variance with Bonferroni’s test. The incidence of High PVC between different conditions was compared using Fisher’s exact test. P<0.05 was considered statistically significant.

### Drug

Olmesartan was kindly provided by Daiichi Sankyo Pharmaceutical Co. (Tokyo, Japan).

## Results

### Effects of Olmesartan on the Development of Cardiomegaly and Contractile Dysfunction in Gα_q_-TG Mice

**Figure 1:**
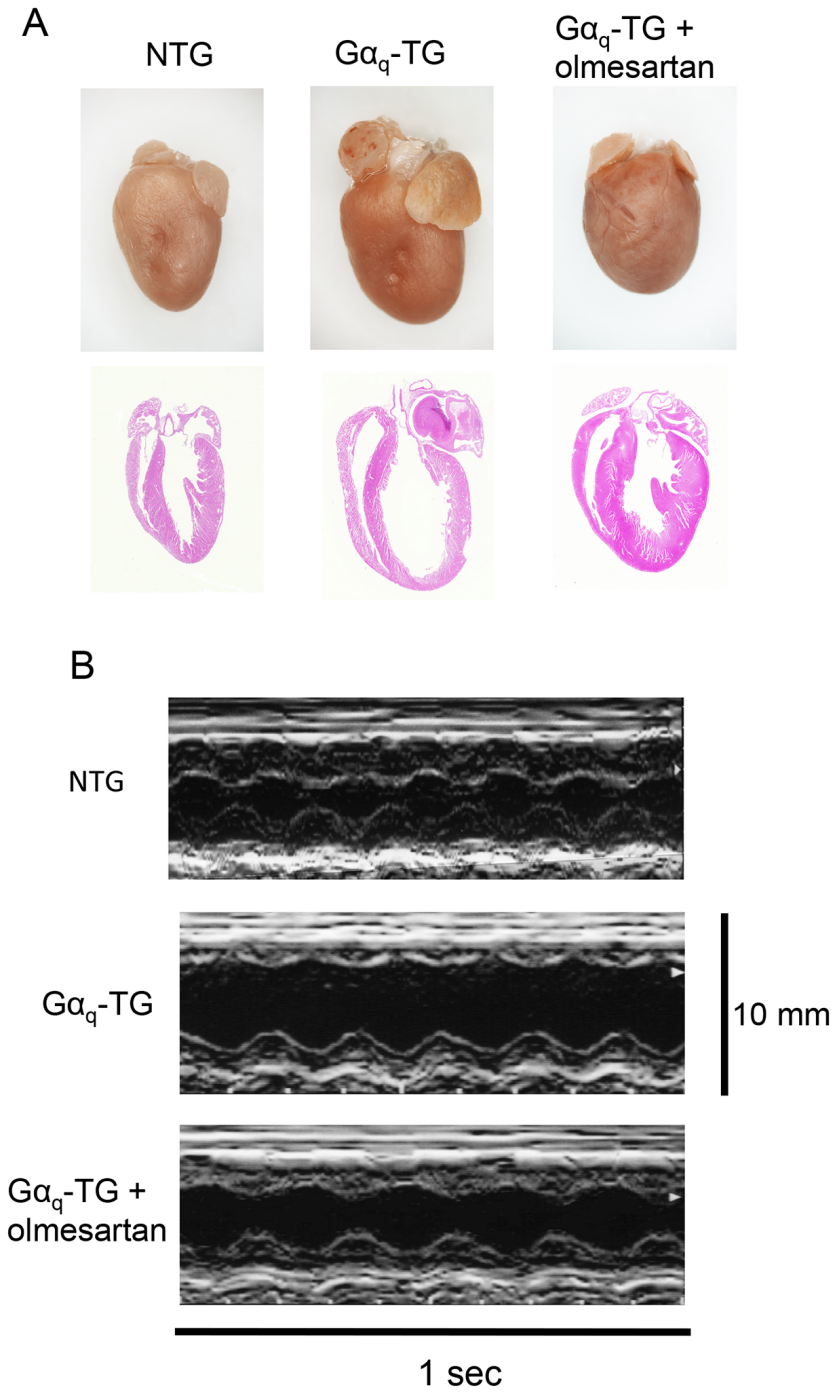
Effects of olmesartan on cardiac morphology and on the left ventricular contractile function. Panel A: Gross examination of a heart and its four-chamber view histology stained with hematoxylin/eosin in NTG, Gα_q_-TG, and Gα_q_-TG+olmesartan mouse hearts. The four-chamber view histology revealed all chambers to be dilated in the vehicle-treated Gα_q_-TG heart compared with those in NTG and olmesartan-treated Gαq-TG hearts. Original magnification: 1.25×. Mice at the age of 32 weeks were used. Panel B: Representative M-mode echocardiograms of NTG, Gα_q_-TG, and Gα_q_-TG+olmesartan mice at the age of 32 weeks.

Effects of chronic administration of olmesartan on cardiac morphology was examined in NTG, vehicle-treated Gα_q_-TG, and olmesartan-treated Gα_q_-TG mice at the age of 32 weeks. All four-chambers were dilated in the vehicle-treated Gα_q_-TG heart compared with those in NTG and olmesartan-treated Gαq-TG hearts (Fig. 1A). The marked cardiomegaly was observed in the vehicle-treated Gα_q_-TG mouse. The heart/body weight ratio increased in vehicle-treated Gα_q_-TG mice compared with that in NTG mice. Olmesartan significantly reduced the ratio in Gα_q_-TG mice (Table 2). The left atrial size**/**tibial length ratio was also increased in vehicle-treated Gα_q_-TG compared with that in NTG hearts. Olmesartan also decreased the ratio in Gα_q_-TG hearts (Table 2). Representative M-mode echocardiograms are shown in Figure 1B. Compared with the NTG mice, vehicle-treated Gα_q_-TG mice showed the markedly reduced LVFS and the increased LVEDd (Fig. 1B and Table 3). Interestingly, olmesartan significantly improved the reduced LVFS and increased LVEDd in Gα_q_-TG mice (Fig. 1B and Table 3).

**Table 2:** General parameters and the incidence of premature ventricular contraction (PVC) in NTG, Gα_q_-TG, and Gα_q_-TG+olmesartan mice.

Parameters	NTG	Gα_q_-TG	Gα_q_-TG+olmesartan
BW (g)	26.5±1.9	27.8±2.0	32.0±1.9
HW (mg)	133±5.5	206±20.4^c^	172±8.9^a^
HW/BW (mg/g)	5.1±0.4	7.5±0.7^b^	5.4±0.5^+^
LA/TL (mm/mm)	0.14±0.06	0.33±0.03^c^	0.19±0.02^$^
PVC (>20 beats/min)	0/10	9/10^c^	0/10^+^

Data are the mean ± SE obtained from 10 mice for each group. ^a^p<0.05, ^b^p<0.01, ^c^p<0.001 vs. NTG, ^+^p<0.01, ^$^p<0.001 vs. values in corresponding parameters of Gα_q_-TG.

**Table 3:** Echocardiographic parameters in NTG, Gα_q_-TG, and Gα_q_-TG+olmesartan mice.

Parameters	NTG	Gα_q_-TG	Gα_q_-TG+olmesartan
IVS (mm)	0.73±0.03	0.62±0.05	0.75±0.07
LVEDd (mm)	2.6±0.2	3.5±0.1^c^	2.8±0.2^+^
LVFS (%)	50.9±2.5	25.4±1.7^c^	44.2±2.5^a,$^

Data are the mean ± SE obtained from 7 mice for each group. ^a^p<0.01, ^c^p<0.001 vs. NTG, ^+^p<0.01, ^$^p<0.001 vs. values in corresponding parameters of Gα_q_-TG. LVEDd, left ventricular end-diastolic dimension; IVS, intraventricular septum.

### Olmesartan-induced Reduction of the Number of Premature Ventricular Contractions (PVCs) in Gα_q_-TG Mice

**Figure 2:**
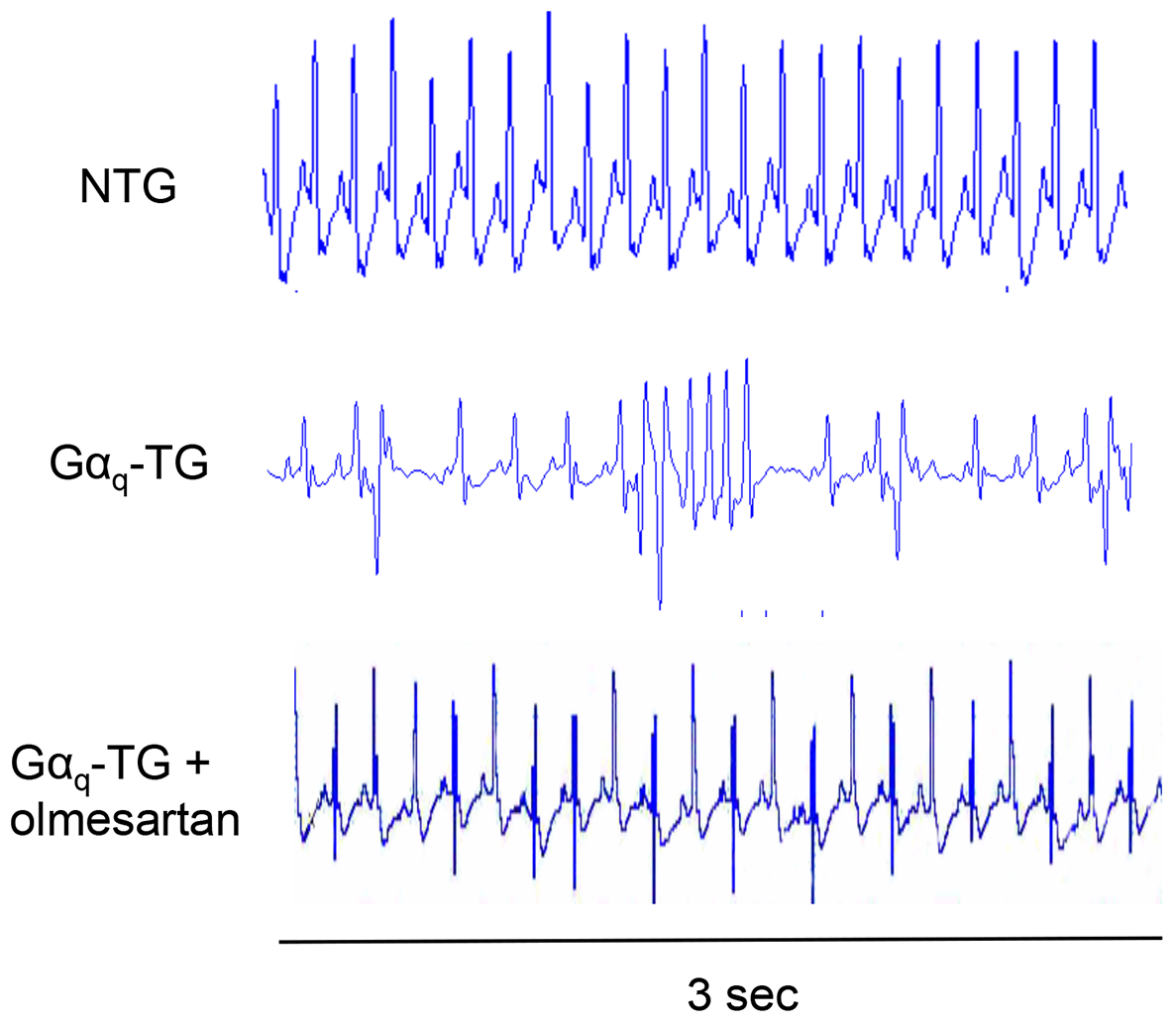
Electrocardiogram (ECG) lead II recordings from NTG, Gα_q_-TG, and Gα_q_-TG+olmesartan mice. The middle ECG shows ventricular arrhythmias recorded from vehicle-treated Gα_q_-TG mice. PVC was frequently observed. In contrast, the upper and lower ECGs recorded from an NTG and olmesartan-treated Gα_q_-TG mouse showed P waves and QRS complexes with regular RR intervals without any arrhythmia, indicating a sinus rhythm. Mice at the age of 32 weeks were used.

Figure 2 shows representative ECGs recorded form anesthetized NTG, vehicle-treated Gα_q_-TG, and olmesartan-treated Gα_q_-TG mice. The middle ECG shows ventricular arrhythmias recorded from vehicle-treated Gα_q_-TG mice. Premature ventricular contraction (PVC) and non-sustained ventricular tachyarrhythmia (VT) were frequently observed. In contrast, the upper and lower ECGs recorded from an NTG- and olmesartan-treated Gα_q_-TG mouse showed P waves and QRS complexes with regular RR intervals without any arrhythmia, indicating a sinus rhythm. Table 2 shows the overall data for ventricular arrhythmias. NTG mice did not induce ventricular arrhythmias such as a high PVC count (more than 20 beats/min). In contrast, a high number of PVCs was observed in 9 of 10 vehicle-treated Gα_q_-TG mice (Table 2). Moreover, non of olmesartan-treated Gα_q_-TG mice induced a high PVC count, indicating a significant reduction of ventricular arrhythmias in olmesartan-treated Gα_q_-TG mice compared with that in vehicle-treated Gα_q_-TG mice.

### Effects of Olmesartan on Changes in Electrocardiogram Parameters in Gα_q_-TG Mice

Overall data for the electrophysiological parameters in NTG, vehicle-treated Gα_q_-TG, and olmesartan-treated Gα_q_-TG mice at 32 weeks of age are shown in Table 4. P, PR, QRS, and QT interval were longer in vehicle-treated Gα_q_-TG mice than in NTG mice. Interestingly, while the prolonged QRS interval was still observed in olmesartan-treated Gα_q_-TG mice compared with NTG mice, the P, PR, and QT intervals were restored to normal levels in olmesartan-treated Gα_q_-TG compared with those in vehicle-treated Gα_q_-TG mice.

**Table 4:** Electrocardiographic parameters in NTG, Gα_q_-TG, and Gα_q_-TG+olmesartan mice.

Parameters	NTG	Gα_q_-TG	Gα_q_-TG+olmesartan
P (msec)	21±1	29±1^b^	22±1^$^
RR (msec)	182±12	218±29	229±19
PR (msec)	49±5	87±8^a^	50±4^&^
QRS (msec)	15±0.6	20±1^a^	21±1^a^
QT (msec)	33±1	43±2^b^	37±1^a,+^

Data are the mean ± SE obtained from 7 mice for each group. ^a^p<0.05, ^b^p<0.001 vs. WT, ^+^p<0.05, ^&^p<0.01, ^$^p<0.001 vs. values in corresponding parameters of vehicle-treated Gα_q_-TG.

### Effects of Olmesartan on Myocardial Fibrosis and the mRNA Expression of Profibrotic Genes in Gα_q_-TG Mice

**Figure 3:**
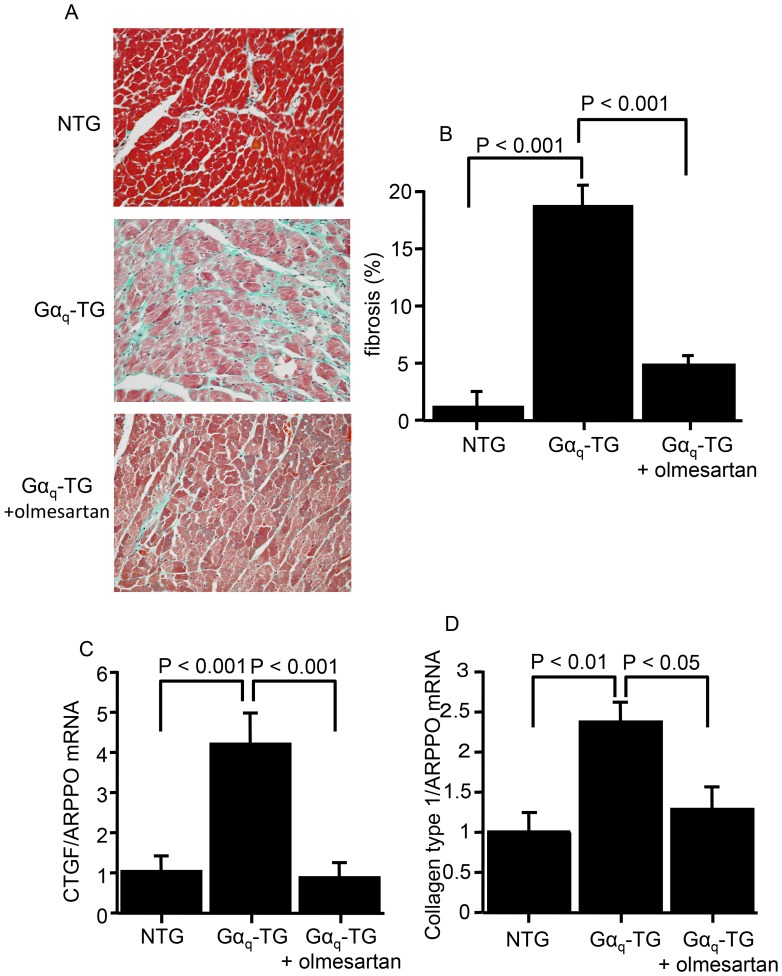
Effects of olmesartan on the left ventricular fibrosis and on connective tissue growth factor (CTGF) and collagen type 1 gene expression. Panel A: Histology of the left ventricle stained with Masson’s trichrome in NTG, Gα_q_-TG, and Gα_q_-TG+olmesartan mice. Original magnification: 40×. Panel B: Comparison of the fibrosis fraction in the left ventricle in NTG, Gα_q_-TG, and Gα_q_-TG+olmesartan mice. Panels C and D: Quantitative analyses of CTGF (C) and collagen type 1 (D) gene expression by real-time reverse transcriptase-polymerase chain reaction (RT-PCR) in NTG, Gα_q_-TG, and Gα_q_-TG+olmesartan hearts. Data for CTGF and collagen type 1 were normalized to those for ARPP0. Data are the mean ± SE obtained from 6 mice for each group.

The effects of chronic olmesartan administration on left ventricular myocardial fibrosis and profibrotic gene expressions of Gα_q_-TG mice at the age of 32 weeks are shown in figure 3. Vehicle-treated Gα_q_-TG hearts induced extensive interstitial fibrosis in the left ventricle compared with that in NTG and olmesartan-treated Gα_q_-TG hearts. The degree of myocardial fibrosis in the left ventricle was significantly greater in vehicle-treated Gα_q_-TG mice compared with that in NTG mice (Fig. 3B). Olmesartan-tretated Gα_q_-TG mice showed the reduced interstitial fibrosis compared with vehicle-treated Gα_q_-TG mice (Fig. 3B). Interestingly, compared with NTG mouse hearts CTGF and collagen type 1 mRNA expression levels were significantly upregulated in vehicle-treated Gα_q_-TG mouse hearts (Fig. 3C and D). Olmesartan significantly decreased the increased expression of those profibrotic genes in Gα_q_-TG hearts (Fig. 3C and D).

### Effects of Olmesartan on Cardiomyocyte Hypertrophy, Fetal Gene Expression and TRPC 6 Channel Protein Levels in Gα_q_-TG Mice

**Figure 4:**
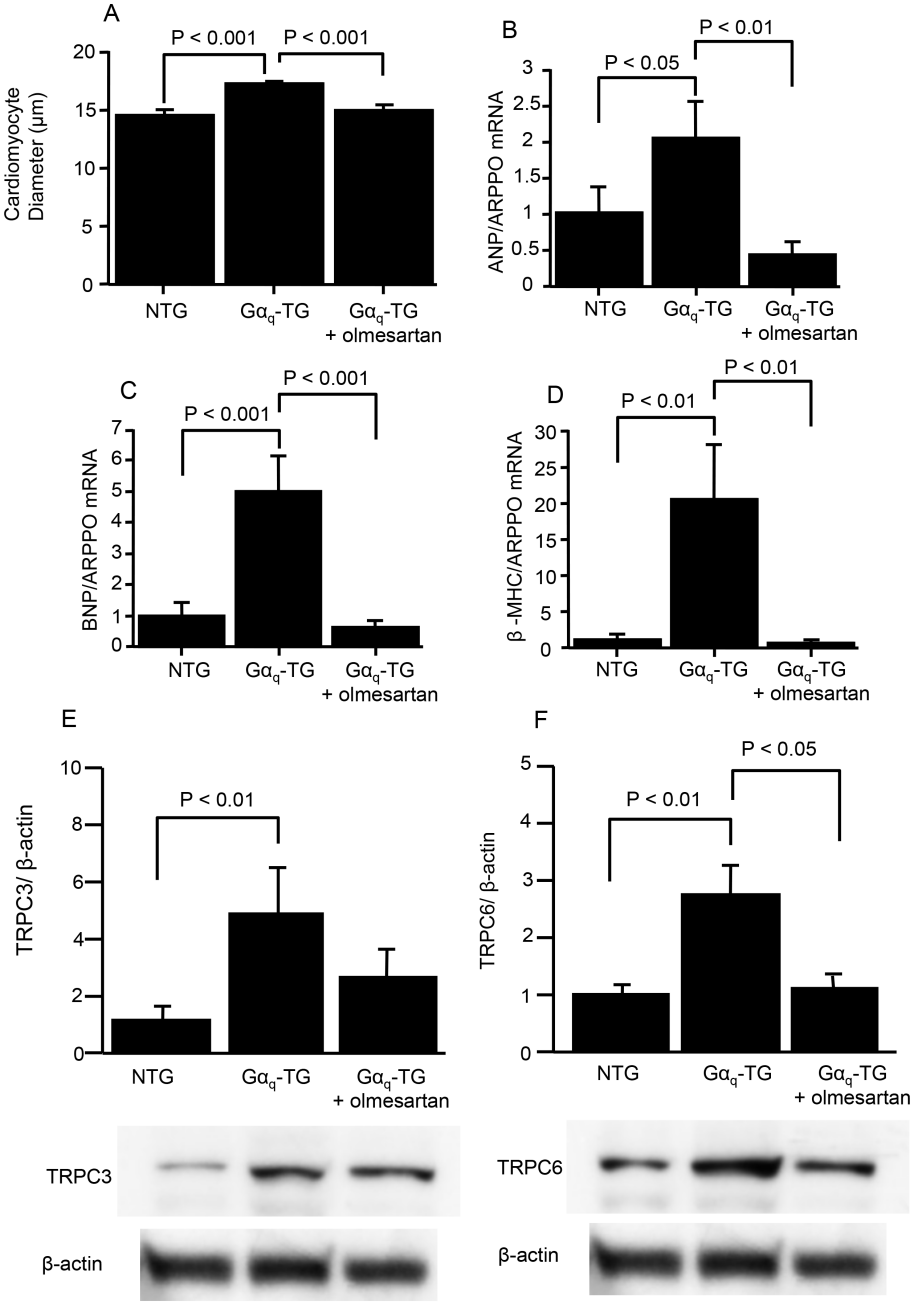
Effects of olmesartan on the left ventricular hypertrophy, on ANP, BNP, and β-MHC gene expression, and on protein expression of canonical transient receptor potential (TRPC) channel isoforms. Panel A: Comparison of cardiomyocyte size in the left ventricle in NTG, Gα_q_-TG, and Gα_q_-TG+olmesartan mice. Panels B–D: Quantitative analyses of ANP (B), BNP (C), and β-MHC (D) gene expression by real-time RT-PCR in NTG, Gα_q_-TG, and Gα_q_-TG+olmesartan hearts. Data for ANP, BNP, and β-MHC were normalized to those for ARPP0. Data are the mean ± SE obtained from 6 mice for each group. Panel E: Expression of TRPC channel isoforms in NTG, Gα_q_-TG, and Gα_q_-TG+olmesartan hearts. TRPC isoform expression was normalized to actin expression and is expressed relative to wt (set at 1). Data are the mean ± SE obtained from 6 mice for each group. ANF, atrial natriuretic factor; BNP, B-type natriuretic peptide; β-MHC, β-myosin heavy chain; ARPP0, acidic ribosomal protein P0. Mice at the age of 32 weeks were used.

The effects of olmesartan on the cardiomyocyte hypertrophy and the mRNA expression of fetal type genes such as ANF, β-MHC, and BNP in Gα_q_-TG mice are shown in figure 4. The cross-sectional diameter of cardiomyocytes in vehicle-treated Gα_q_-TG mice was longer than that in NTG mice (Fig. 4A). The increased cross-sectional diameter was significantly decreased in olmesartan-treated Gα_q_-TG mice (Fig. 4A). Moreover, the mRNA expression levels of ANF, BNP, and β-MHC were significantly upregulated in Gα_q_-TG hearts compared with that in NTG mouse hearts (Figs. 4B, C, and D). The increased gene expression of ANF, BNP, and β-MHC was decreased in olmesartan-treated Gα_q_-TG hearts (Figs. 4B, C, and D). Recent studies have suggested that the activation of TRPC channels plays important roles in the generation of cardiac hypertrophy and cardiac arrhythmia induction [4], [16]. Moreover, TRPC3 and 6 protein expression levels were increased in Gα_q_-TG mouse hearts [4]. Therefore, we examined the effects of olmesartan on the protein expression of TRPC3 and 6 channels in Gα_q_-TG hearts. Compared with NTG hearts, the vehicle-treated Gα_q_-TG hearts exhibited the increased TRPC 3 and 6 protein levels (Figs. 4E and F). The increased expression of TRPC 6 protein was decreased in olmesartan-treated Gα_q_-TG mouse hearts (Fig. 4F).

### Effects of Olmesartan on Angiotensin Converting Enzyme (ACE) and Angiotensin II Type 1 (AT_1_) Receptor Protein Expression in Gα_q_-TG Mice

**Figure 5:**
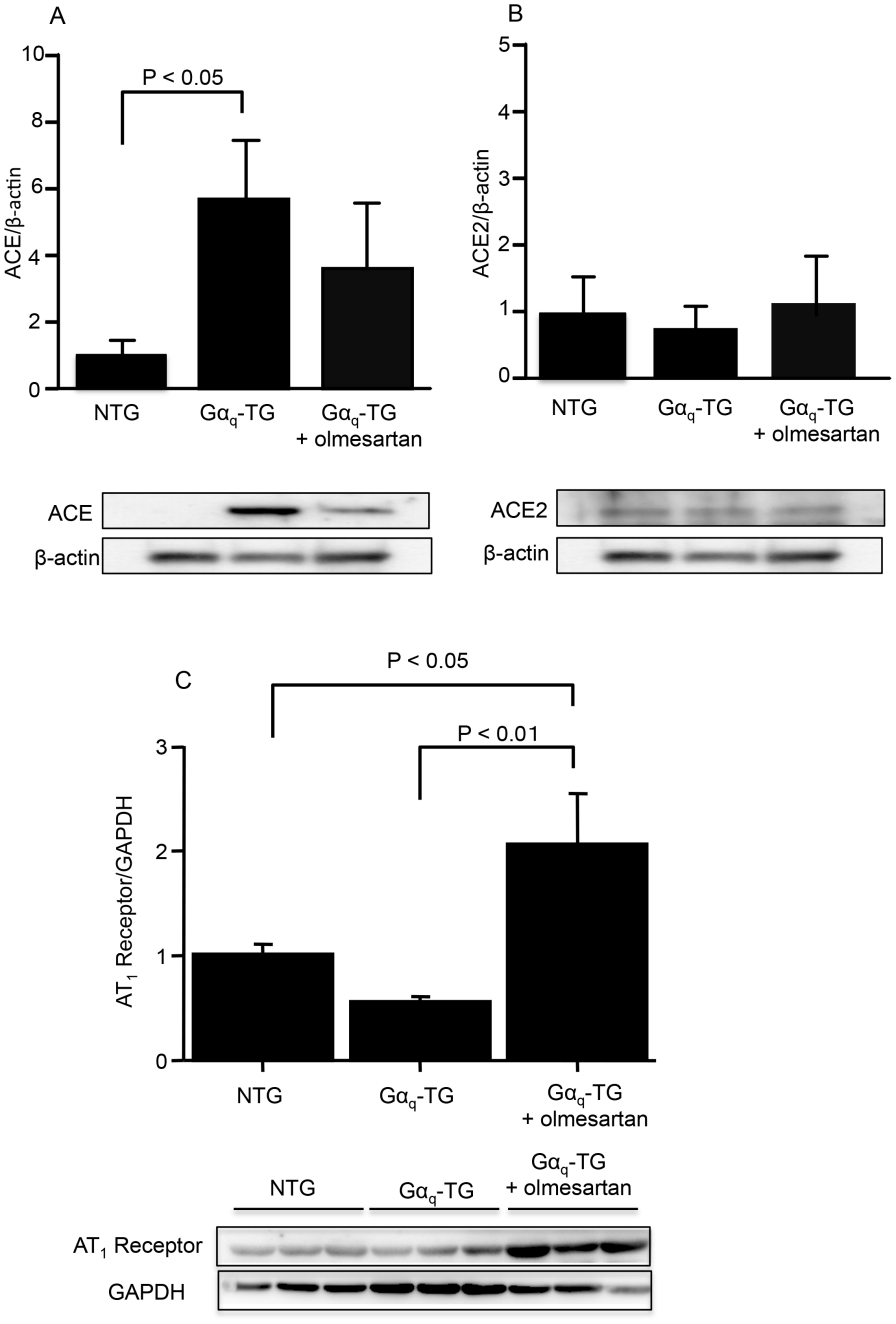
Effects of olmesartan on protein expression of angiotensin converting enzyme (ACE) isoforms and angiotensin II type 1 (AT_1_) receptor. Expression of ACE (A), ACE2 (B), and AT_1_ receptor (C) in NTG, Gα_q_-TG, and Gα_q_-TG+olmesartan hearts. ACE isoform expression was normalized to actin expression and is expressed relative to NTG (set at 1). AT_1_ receptor was normalized to GAPDH and is expressed relative to NTG (set at 1). Data are the mean ± SE obtained from 6 mice for each group. Mice at the age of 32 weeks were used.

We examined the protein expression levels of ACE, ACE2, and AT_1_ receptor in NTG, vehicle-treated Gα_q_-TG, and olmesartan-treated Gα_q_-TG mice at the age of 32 weeks. The level of ACE but not ACE2 was significantly increased in Gα_q_-TG hearts compared with that in NTG hearts (Figs. 5A and B). Olmesartan tended to decrease the increased expression of ACE in Gα_q_-TG mouse hearts (Fig. 5A). The level of AT_1_ receptor was not changed in Gα_q_-TG hearts compared with that in NTG hearts (Fig. 5C). Olmesartan significantly increased the expression of AT_1_ receptor in Gα_q_-TG mouse hearts (Fig. 5C).

### Effects of Olmesartan on Ventricular Monophasic Action Potential (MAP) in Gα_q_-TG Mice

**Figure 6:**
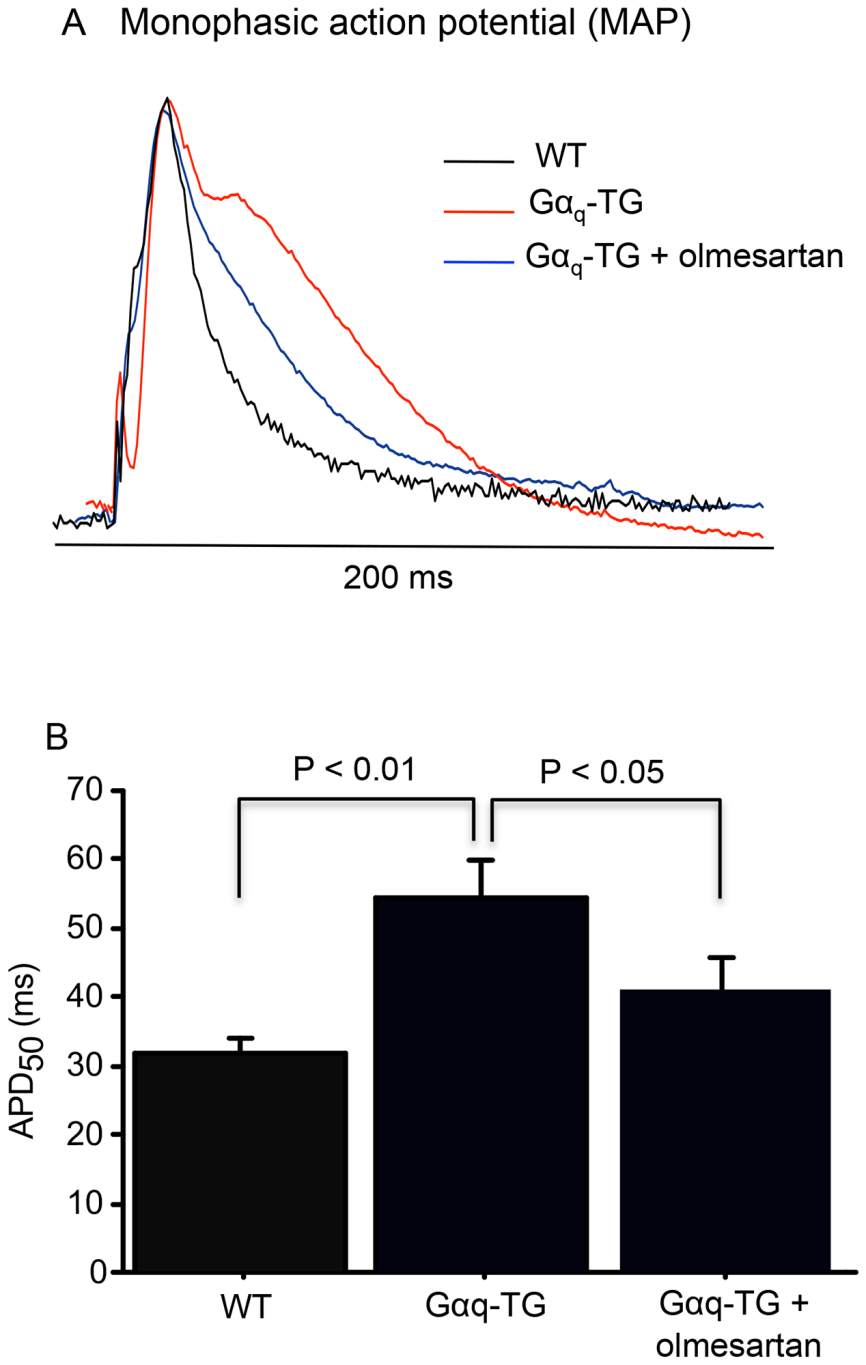
Effects of chronic olmesartan treatment on ventricular monophasic action potential (MAP) duration. Panel A: Representative examples of MAPs recorded from the posterior left ventricle in a Langendorff-perfused in NTG, Gα_q_-TG, and Gα_q_-TG+olmesartan hearts during steady state pacing at a cycle length of 200 msec. Panel B: Overall data of MAP duration in in NTG, Gα_q_-TG, and Gα_q_-TG+olmesartan hearts.

Figure 6A showed examples of left ventricular MAPs in Langendorff-perfused NTG, Gα_q_-TG, and olmesartan-treated Gα_q_-TG mouse heart. The MAP duration prolonged in the Gα_q_-TG heart compared with that in the NTG and olmesartan-treated Gα_q_-TG mouse hearts. The overall data demonstrated that the chronic administration of olmesartan significantly shortened the ventricular MAP duration in Gα_q_-TG hearts.

## Discussion

In this study, we found that ACE but not ACE2 and AT_1_ receptor protein expression was increased in vehicle-treated Gα_q_-TG mouse hearts. Moreover, chronic administration of olmesartan for 26 weeks prevented the progression of heart failure and ventricular arrhythmia in Gα_q_-TG mice. We also found that olmesartan inhibited ventricular interstitial fibrosis and ventricular myocyte hypertrophy in Gα_q_-TG. CTGF, collagen type 1, ANP, BNP, and β-MHC gene expression was increased in vehicle-treated Gα_q_-TG. Olmesartan significantly decreased the expression of these genes in Gα_q_-TG mice. Electrocardiogram demonstrated that premature ventricular contraction (PVC) was frequently observed in 9 of 10 vehicle-treated Gα_q_-TG but in none of 10 olmesartan-treated Gα_q_-TG. These results suggest that angiotensin II type 1receptor activation plays crucial roles in cardiac remodeling and ventricular arrhythmia in Gα_q_-TG mice.

Clinical and experimental studies have demonstrated that the G_q_-phosphoinositide signaling pathway plays important roles in the development of cardiac hypertrophy and heart failure [17]–[21]. It is well known that several bioactive factors such as angiotensin, endothelin, and norepinephrine activate the cardiac G_q_-phosphoinositide signaling pathway. Our previous study showed that transient expression of a constitutively active mutant of Gα_q_ in hearts of transgenic mice is sufficient to induce cardiac hypertrophy and dilatation. In fact, after the initiating stimulus of the transgenic constitutively active Gα_q_ was not detected the cardiac hypertrophy and dilatation continued to progress [1]. We showed that the multiple and persistent changes in signal transduction pathways maintained cardiac pathological changes initiated by early transient expression of constitutively active Gα_q_ [1], [5]. It is well known that the renin-angiotensin system, which increases the level of DAG, plays a critical role in the development of cardiac hypertrophy and HF [6]–[8]. In addition, cardiac renin-angiotensin system activation (i.e. local) is important in the development of cardiac hypertrophy [22]. Moreover, increased cardiac tissue ACE is known to play important roles in cardiac remodeling [23]. In this study, the protein expression of ACE was increased significantly in Gα_q_ TG mouse hearts compared with that in NTG mouse hearts. In addition, left ventricular myocyte hypertrophy was observed and olmesartan significantly inhibited it (Fig. 4A), which was associated with the prevention of HF and ventricular arrhythmia induction in Gα_q_-TG mice (Fig. 4A). Moreover, mRNA expression of ANF, β-MHC, and BNP was significantly upregulated in Gα_q_-TG hearts compared with that in NTG mouse hearts and decreased by olmesartan in Gα_q_-TG hearts (Figs. 4B, C, and D). These results suggest that transient Gα_q_ activation causes activation of the local renin-angiotensin system, leading to progressive heart failure and ventricular arrhythmias in Gα_q_-TG mice. These findings suggest that the cardiac renin-angiotensin system plays an important role in the development of cardiac hypertrophy and heart failure, even if the initiating stimulus of cardiac Gα_q_ activation does not result from angiotensin II type I (AT_1_) receptor stimulation.

Several studies have demonstrated that cardiac remodeling is associated with increases in AT_1_ receptor protein expression [24]–[26]. Moreover, olmesartan suppressed cardiac AT_1_ receptor levels in hypertensive rats [24]. In this study, the protein expression levels of AT_1_ receptor were not changed in Gα_q_-TG hearts compared with those in NTG hearts (Fig. 5C). Moreover, olmesartan significantly increased the expression of AT_1_ receptor in Gα_q_-TG mouse hearts (Fig. 5C). The reason for the discrepancy between the previous and present results is uncertain. In fact, cardiac dysfunction is severe in this Gα_q_-TG mouse compared with that in animals used in previous studies [24], [26]. Moreover, the duration of olmesartan treatment was much longer in this study than in the previous study [24]. Those differences may explain the discrepancy. In any case, our present results suggest that AT_1_ receptor activation plays important roles in the development of heart failure and ventricular arrhythmias in this model.

It is known that myocardial ACE is a possible substrate for cardiac fibrosis [23]. In this study, the protein expression of ACE was increased significantly in Gα_q_ TG mouse hearts compared with that in NTG mouse hearts. Moreover, the left ventricular fibrosis and mRNA expression of CTGF and collagen type I were also significantly increased in Gα_q_-TG mouse hearts. Olmesartan decreased the increased left ventricular fibrosis and the mRNA expression of CTGF and collagen type I, suggesting that the renin-angiotensin system participates in the development of cardiac fibrosis in this model. Importantly, together these findings suggest that the cardiac renin-angiotensin system plays an important role in the development of cardiac hypertrophy, fibrosis and heart even if the initiating stimulus of cardiac Gα_q_ activation does not result from AT_1_ receptor stimulation.

It has been shown that mechanical stress activates AT_1_ receptor independently of angiotensin II, and this activation can be inhibited by an inverse agonist of the AT_1_ receptor [27]–[28]. Our previous study demonstrated that the left ventricular end-diastolic pressure was increased in Gα_q_-TG compared with that in NTG mice [2], suggesting that mechanical stretching of the myocardium was induced in Gα_q_-TG mice, leading to activation of AT_1_ receptors. Recent study has demonstrated that olmesartan has strong inverse agonist activities against the constitutively active AT_1_ receptor and the stretch-induced activation of AT_1_ receptor, respectively [28]. Therefore, olmesartan induced inhibition of ventricular myocyte hypertrophy and interstitial fibrosis in Gα_q_-TG may be caused in part through inverse agonistic action.

In this study, chronic administration of olmesartan prevented the progression of heart failure and ventricular arrhythmia in Gα_q_-TG mice. In fact, electrocardiogram demonstrated that PVC was frequently (more than 20 beats/min) observed in 9 of 10 vehicle-treated Gα_q_-TG mice but in none of 10 olmesartan-treated Gα_q_-TG mice. In addition, the QT interval was significantly shorter in olmesartan-treated Gα_q_-TG than in vehicle-treated Gα_q_-TG mice. Moreover, the MAP duration was also significantly shorter in olmesartan-treated Gα_q_-TG than in vehicle-treated Gα_q_-TG mice. It is well known that ventricular arrhythmias are common in heart failure. However, a recent study demonstrated that chronic angiotensin II stimulation in the heart directly induced QT prolongation through down-regulation of potassium channels, [29] which can induce triggered activity, leading to the production of PVC. Moreover, a recent study clearly demonstrated that AT_1_ receptor signaling in the heart directly contributed to the increased arrhythmogenicity in cardiac hypertrophy [30]. In fact, our previous study demonstrated that early-after depolarization by the prolongation of action potential duration caused triggered activity. Therefore, in addition to improvement of heart failure olmesartan might directly inhibit PVC induction because of the shortening of action potential duration. We previously demonstrated that the protein levels of TRPC3 and 6 are increased in Gα_q_-TG hearts [4] and suggested that the activation of TRPC channels participates in the generation of cardiac arrhythmia induction. Interestingly, olmesartan decreased the increased expression of TRPC 6 in Gα_q_-TG mouse hearts (Fig. 4F) in this study, suggesting that AT_1_ receptor activation contributes to an increase in TRPC6 expression, leading to ventricular arrhythmia induction.

## Supporting Information

Table S1 **Primers used in this study.**
(XLS)
